# The Field-Dependent Magnetic Viscosity of FeNdB Permanent Magnets

**DOI:** 10.3390/ma17010243

**Published:** 2024-01-02

**Authors:** Thomas Kresse, Gerhard Martinek, Gerhard Schneider, Dagmar Goll

**Affiliations:** Materials Research Institute, Aalen University, 73430 Aalen, Germany; gerhard.martinek@hs-aalen.de (G.M.); gerhard.schneider@hs-aalen.de (G.S.)

**Keywords:** NdFeB, magnetic viscosity, magnetic aging, magnetic hysteresis loop, coercivity, demagnetization, permanent magnets

## Abstract

The time-dependent decrease of the magnetic polarization of magnet materials in the presence of an opposing field is well known as the magnetic viscosity or magnetic aftereffect. In previous studies, magnetic viscosity was usually measured in fields when in the vicinity of coercivity $H_{\mathrm{cJ}}$, and this was conducted in order to understand the coercivity mechanism in magnetic materials. In this study, the magnetic viscosity of commercial FeNdB magnets is determined at opposing fields weaker than $H_{\mathrm{cJ}}$ and at different temperatures in the range from 303 to 433 K (i.e., from 30 to 160 °C) by means of a vibrating sample magnetometer (VSM). As a result, the parameter $S_{v}$, which describes the magnetic viscosity in the material, was found to increase with increases in the opposing field. Furthermore, both the parameter $S_{v}$ and its dependence on the temperature were found to correlate with the coercivity $H_{\mathrm{cJ}}$ of the material. Also, a difference with regard to the parameter $S_{v}$ for the materials measured in this study with similar magnetic properties, but which had undergone different types of processing, could not be found. Knowledge of the field- and temperature-dependent behavior of the magnetic viscosity of FeNdB magnets allows for better estimations over the lifetime of a component under operating conditions with respect to the magnetic losses in FeNdB magnets that are used in electric components.

## 1. Introduction

These days, the automotive sector is one of the largest fields of application for permanent magnets [1,2]. Magnets are used for many different applications within an automobile (e.g., oil pumps, alternators, starters, air conditioning, and windshield wipers). Traditionally, hard ferrites were the most widely installed magnets in cars due to their very low material costs [1]. However, against the backdrop of advancing electromobility, FeNdB magnets have been increasingly used in the automotive sector in recent years, mainly in the traction motors of both battery and hybrid electric vehicles [1,3,4]. The reason for this is that FeNdB magnets, in contrast to hard ferrites, allow high torques and power densities to be realized in such motors [1], and they can achieve this with a very compact design at the same time because of their high maximum energy density [2,5]. However, once the magnets have been installed in the component, they cannot usually be magnetized again if they partially lose their performance in the presence of an external opposing magnetic field or temperature. In order to guarantee a maximum lifetime for the corresponding electric component, the thermally activated aging of magnets in an applied opposing magnetic field has to be considered in the machine design and in the selection of the used hard magnetic materials. Particularly in the case of FeNdB magnets, it is common practice to choose higher and more expensive magnet grades with regard to coercivity and thus magnetic stability. This ensures that the guaranteed power of the machine is preserved in spite of the magnetic loss over the lifetime of the component. The thermally activated aging of magnets, also known as the magnetic viscosity or magnetic aftereffect, has been investigated for several magnetic materials in previous studies [6,7,8,9,10,11,12,13]. However, the main goal of most of these studies was to better understand the basic coercivity mechanism in the investigated material [14,15]. Hence, the magnetic fields at which the magnetic viscosity measurements were carried out were mainly in the range between the coercivity $H_{\mathrm{cJ}}$ and the so-called “knee” of the demagnetization curve of the magnet [9,10,12,15,16], which is where the majority of the demagnetization processes occur. Other studies have focused on the magnetic loss over long time periods without applied fields [17,18]. On the other hand, the field range between remanence and the “knee” has not thus far been investigated with regard to magnetic viscosity. The reason for this is that, at magnetic fields that are far from the parameter coercivity $H_{\mathrm{cJ}}$, the time-dependent effects are comparatively weak [15] and are thus of minor relevance for the investigation of the coercivity mechanism. In practice, a magnet that is used in a component is supposed to be subject to external fields that are lower than the fields at which the magnet mainly demagnetizes. Therefore, the knowledge of the aging behavior in this field range is crucial for estimating the thermally activated magnetic loss of the magnet materials in magnetic fields that are generally used in industrial applications. In this study, the thermally activated aging of commercial, sintered FeNdB magnets of different magnet grades was investigated at both the prevailing temperatures and external magnetic fields that are used in electrical machines. This allowed for a better estimation of the magnetic aging under real operating conditions. As a result, more suitable and cost-efficient magnetic materials could be chosen for the machine design.

## 2. Theoretical Background

Permanent magnets are magnetically metastable systems [7,19]. At a certain type of opposing magnetic field, a demagnetization of individual metastable states (i.e., domains) occurs in materials due to the thermal activation that takes place over certain energy barriers. The decrease in the magnetic polarization *J* at a given opposing field has been observed as a logarithmic function of time *t* [7,14,19] as follows:

(1) $$J\left(t\right)=J\left(t_{0}\right)-\mu_{0}\;S\;\ln\left(t/t_{0}\right).$$

The slope of the logarithmic decrease in *J* is defined as the magnetic viscosity *S* while $t_{0}$ and $\mu_{0}$ are the starting time of the demagnetization and the vacuum permeability, respectively. Due to the fact that the demagnetized states in the material hardly change their magnetization direction again, the time-dependent decrease in polarization can be interpreted as the irreversible part of the demagnetization of the material when in an opposing magnetic field. Néel [20,21] suggested that the effect of thermal activation on the magnetic polarization can be expressed by an additional “effective” fluctuation field

(2) $$\Delta H=-S_{v}\;\ln\left(t/t_{0}\right),$$

which would have the same effect on the magnetic polarization as follows:

(3) $$J\left(t\right)=J\left(t_{0}\right)-\mu_{0}\;\chi^{\mathrm{irr}}\;S_{v}\;\ln\left(t/t_{0}\right),$$

where $S_{v}$ is the so-called magnetic viscosity parameter, while $\chi^{\mathrm{irr}}$ is the irreversible susceptibility. The comparison of Equations (1) and (3) leads to

(4) $$S_{v}=S/\chi^{\mathrm{irr}}.$$

For magnetic samples with a demagnetization factor N≠0, the parameter $S_{v}$ has to be slightly adjusted in order to consider the internal demagnetization field of the sample [8]:

(5) $$S_{v}=\frac{S}{\chi_{\mathrm{ext}}^{\mathrm{irr}}}\;\left(1-N\;\chi_{\mathrm{ext}}^{\mathrm{rev}}\right),$$

where $\chi_{\mathrm{ext}}^{\mathrm{rev}}$ and $\chi_{\mathrm{ext}}^{\mathrm{irr}}$ are the reversible and irreversible susceptibility of the material, respectively, in regard to the applied external field $H_{\mathrm{ext}}$.

## 3. Materials and Methods

For this study, four commercially available anisotropic sintered permanent magnets (based on FeNdB with different compositions and thus different magnetic properties like coercivity $H_{\mathrm{cJ}}$) were selected, as shown in Table 1. In detail, a material without any heavy rare-earth elements (HREEs) like Tb or Dy was chosen (M1), as well as a magnetic material with a high amount of HREEs and a larger grain size (M4). The materials M2 and M3 both contained a small amount of HREEs and exhibited similar magnetic properties. However, while material M2 was sintered conventionally (as were the materials M1 and M4), in case of material M3, the HREE amount was introduced into the sintered material by a subsequent grain boundary diffusion process (GBDP). The chosen materials therefore represent a suitable selection that roughly covers the available commercial FeNdB magnets.

The magnetic measurements were carried out by means of a vibrating sample magnetometer (VSM) with a Quantum Design (model PPMS-9). For this purpose, small cuboid pieces (with a length and width between 2.5 and 4.5 mm, and a height between 0.45 and 0.65 mm) were cut out of the selected magnets and were polished using ethanol and abrasive paper.

For the determination of the magnetic viscosity, VSM measurements at the temperatures of 303, 343, 373, 403, and 433 K were carried out using the oven option supplied with the VSM. The magnetic viscosity parameter $S_{v}$ for each material and temperature was determined by means of the so-called constant-field method using Equation (5). First, the demagnetization curve $J(H_{\mathrm{ext}})$ was measured, which allowed for calculating the field-dependent total susceptibility $\chi_{\mathrm{ext}}^{\mathrm{tot}}=\chi_{\mathrm{ext}}^{\mathrm{rev}}+\chi_{\mathrm{ext}}^{\mathrm{irr}}=1/\mu_{0}\;\left(dJ/dH_{\mathrm{ext}}\right)$ from the slope of the measured demagnetization curve.

**Figure 1:**
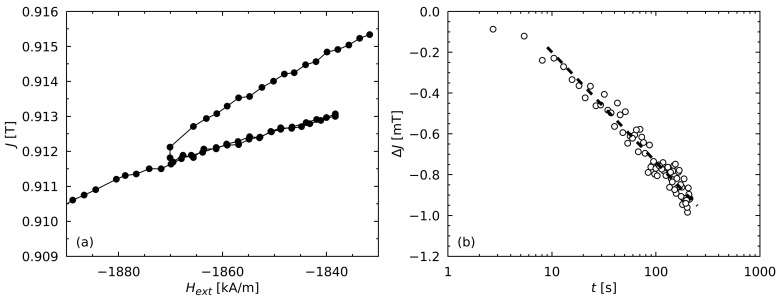
VSM measurements for the determination of the magnetic viscosity parameter $S_{v}$ at T=303K and $H_{\mathrm{ext}}=-1870\;\mathrm{kA}/m$ ($\mu_{0}\;H_{\mathrm{ext}}=-2.35\;T$) for the material M4. (**a**) Partial loop of the demagnetization curve starting at $H_{\mathrm{ext}}=-1870\;\mathrm{kA}/m$. The slope of the partial loop is $\mu_{0}\;\chi_{\mathrm{ext}}^{\mathrm{rev}}$ with $\chi_{\mathrm{ext}}^{\mathrm{rev}}=0.033\pm 0.001$. (**b**) Polarization decrease ΔJ caused by the thermally-activated demagnetization. After a certain time $t_{0}$ the polarization *J* decreases logarithmically with time *t*. The slope of the logarithmic decrease of *J* (dashed line) is $\mu_{0}\;S$ with S=(184±8)A/m.

The measurement of the reversible susceptibility $\chi_{\mathrm{ext}}^{\mathrm{rev}}$ was realized by measuring the partial loops at certain field strengths along the demagnetization curve $J(H_{\mathrm{ext}})$ (Figure 1a). By way of an analogy with $\chi_{\mathrm{ext}}^{\mathrm{tot}}$, $\chi_{\mathrm{ext}}^{\mathrm{rev}}$ was calculated at a selected field from the slope of the corresponding partial loop at this field. The field-dependent value $\chi_{\mathrm{ext}}^{\mathrm{irr}}$ could then be calculated from the difference of $\chi_{\mathrm{ext}}^{\mathrm{tot}}$ and $\chi_{\mathrm{ext}}^{\mathrm{rev}}$.

For the determination of the magnetic viscosity *S*, the sample was at first fully magnetized at an applied field $H_{\mathrm{ext}}$ of 7162 kA/m ($\mu_{0}\;H_{\mathrm{ext}}=9\;T$). Afterward, the applied field was reduced to the selected field strength by a linear approach using a field sweep rate of 0.8 kA/(m s) without overshooting the target field significantly (less than 20 A/m). The temporal decrease in the polarization was measured for about 180 s as shown exemplarily in Figure 1b. The logarithmic time dependence of the polarization decrease started after a certain time $t_{0}$ due to the low overshooting and the relaxations occurring during the approach to the selected field at a finite speed. The viscosity *S* was determined from the slope of the measurement points that showed a time–logarithmic decrease according to Equation (1). As only the measuring points of the logarithmic decay were considered for the determination of *S*, a value of $t_{0}=1\;s$ was set for the starting time in Equation (1). Subsequently, the applied field was further reduced to the next selected field strength, whereby the distance between the selected field strengths was chosen in such a way that the time-dependent decrease in polarization at a certain field strength did not affect the temporal measurement at the next chosen field strength.

To determine the demagnetization factor *N*, which is necessary for the calculation of the internal field $H_{\mathrm{int}}=H_{\mathrm{ext}}-N\;J\left(H_{\mathrm{ext}}\right)/\mu_{0}$, a rough estimate was first made via a theoretical calculation that was performed by [22] based on the rough dimensions of the samples. Due to the fact that the theoretical model proceeds on a simplified assumption (a homogeneous magnetization within the whole cuboid sample) and that the geometries of the samples differed distinctly from ideal cuboid shapes, the calculated values for *N* had to be adjusted to lower values in order to avoid overshooting in the steepest part of the demagnetization curves $J(H_{\mathrm{int}})$. For the investigated samples, the adjusted values for *N* were between 0.07 and 0.12, which is 20 to 30% lower than the theoretical values. Due to these low values for *N*, the uncertainty for $H_{\mathrm{int}}$ was less than 25 kA/m for an assumed uncertainty of 20% for *N*.

As a reference value for the user-relevant internal field $H_{\mathrm{int}}$ of the magnet, the demagnetizing field $H_{D5}$ was chosen. This parameter was defined as the field strength of the internal field at which the magnet exhibits an irreversible loss of 5% of its magnetic moment and is a quantitative measure for the magnetic stability of a magnet against an opposing external field [23]. In practice, the magnets in electrical machines are not subjected to opposing fields stronger than $H_{D5}$, which was subsequently selected as the upper limit for opposing fields in this study.

## 4. Results

**Figure 2:**
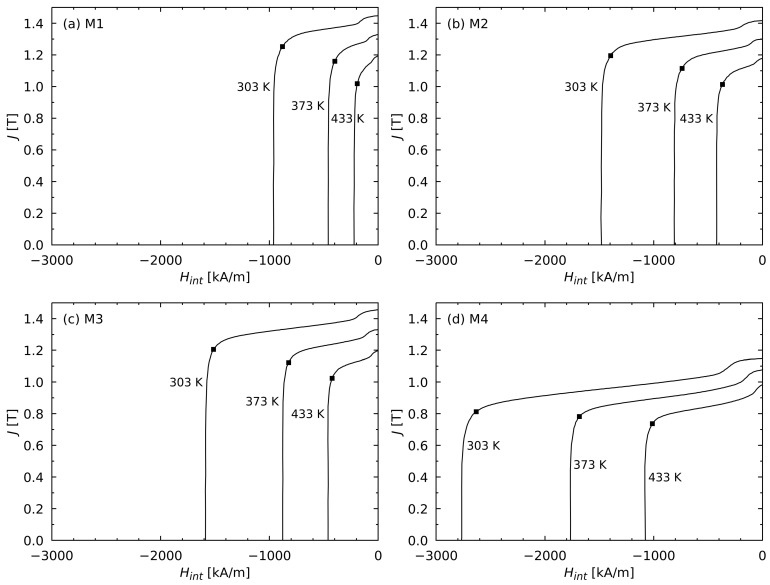
Demagnetization curves of the investigated FeNdB magnets at selected temperatures. The respective positions for the demagnetizing field $H_{D5}$ were determined in accordance with [23], and they are marked with black squares. The decrease in polarization at the right side of the curves was caused by the demagnetization of the areas at the surface that exhibited a strongly reduced coercivity compared to those of the bulk type [24,25,26].

In Figure 2, the demagnetization curves of the investigated FeNdB magnets for the selected temperatures are shown. Table 2 shows the demagnetizing field $H_{D5}$, which was determined in accordance with [23] for all the investigated materials at the selected temperatures.

**Figure 3:**
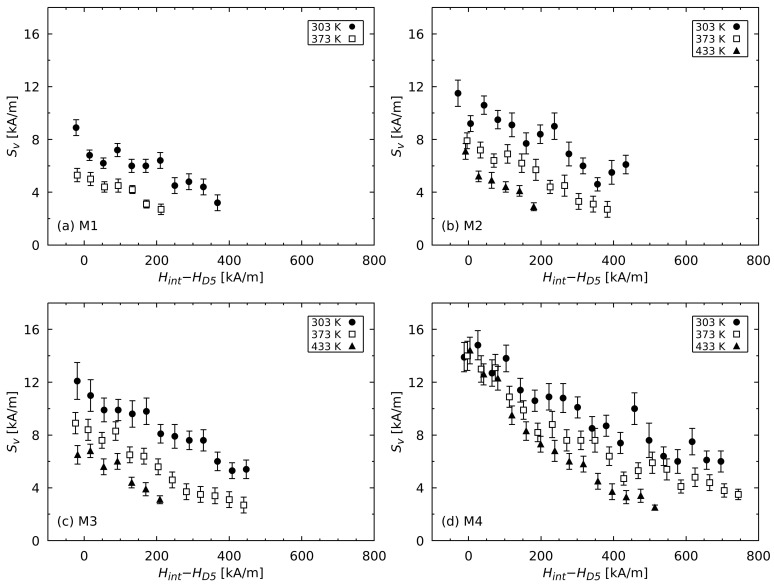
Magnetic viscosity parameter $S_{v}$, which is dependent on the internal magnetic field $H_{\mathrm{int}}$ with regard to the demagnetizing field $H_{D5}$ for selected temperatures.

In Figure 3, the magnetic viscosity parameter $S_{v}$, which is dependent on the internal magnetic field, is shown for the selected temperatures. For all the measured materials, $S_{v}$ increased with an increasing opposing field up to $H_{D5}$. At 303 K, the determined values of $S_{v}$ were in the range between 8 and 16 kA/m, whereby those values were calculated from a linear regression of the last $H_{\mathrm{int}}$ values. In the case of the HREE-free material M1, the magnetic properties (e.g., $H_{\mathrm{cJ}}$) at 433 K were very weak and led to too few measurements at the possible field strengths of the applied field $H_{\mathrm{ext}}$; as such, no measurements of the magnetic viscosity at this temperature were carried out.

**Figure 4:**
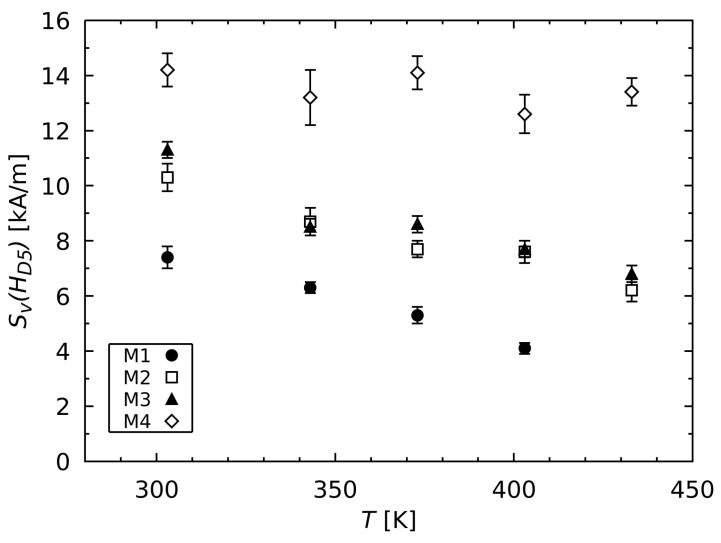
The magnetic viscosity parameter $S_{v}$ at the demagnetizing field $H_{D5}$, which is dependent on the temperature *T*. The values were determined by fitting the corresponding $S_{v}\left(H_{\mathrm{int}}\right)$ values.

The parameter $S_{v}$ at the demagnetizing field $H_{D5}$ decreases with increasing temperature, as shown in Figure 4. However, the decrease in $S_{v}$ varies in intensity for the measured samples, as shown in Table 3. Particularly in case of the high coercive material M4, the value $S_{v}\left(H_{D5}\right)$ only decreased very slightly with increasing temperature. However, the temperature-dependent decrease in $S_{v}$ for this material was stronger at the weaker internal fields $H_{\mathrm{int}}$. On the other hand, the value $S_{v}\left(H_{D5}\right)$ decreased most for the low coercive magnetic material M1 in the measured temperature range. The materials M2 and M3 exhibited the same magnetic behavior regarding both the amount of $S_{v}$ and its temperature dependence. It can also be seen in Figure 4 that the value $S_{v}\left(H_{D5}\right)$ decreased with decreasing coercivity $H_{\mathrm{cJ}}$ at a given temperature in the entire temperature range measured.

## 5. Discussion

In previous studies, the magnetic viscosity parameter $S_{v}$ was usually determined at applied magnetic fields between the coercivity $H_{\mathrm{cJ}}$ and the so-called “knee” of the demagnetization curve [9,10,15,16]. In this magnetic field range, $S_{v}$ was observed to be constant and thus field-independent. On the other hand, Volegova et al. [27] determined the parameter $S_{v}$ for a rapidly quenched FeNdB material at applied fields weaker than $H_{\mathrm{cJ}}$. In their work, an increase in $S_{v}$ with stronger applied fields was observed. In the region of the demagnetizing field $H_{D5}$, the $S_{v}$ values between 12 and 20 kA/m were determined for the stoichiometric compositions of FeNdB. Those results were therefore in good agreement with the results in this study.

For all the measured samples, the value $S_{v}\left(H_{D5}\right)$ increased with increases in coercivity $H_{\mathrm{cJ}}$ at a given temperature. An analogous correlation was observed in the case of $S_{v}\left(H_{\mathrm{cJ}}\right)$ for different magnetic materials in previous studies [6,28]. Moreover, the materials M2 and M3 showed the same behavior regarding the amount of the parameter $S_{v}$, as well as in both its temperature and field dependence despite the different processing methods. Therefore, the extent of the magnetic viscosity mainly depends on the coercivity of the sintered FeNdB magnet, regardless of whether a GBDP is applied or not.

The temperature dependence of magnetic viscosity was also investigated in previous studies. The parameter $S_{v}$ can alternatively be expressed by the following formula [8,14,15,29]:

(6) $$S_{v}=\frac{k_{B}\;T}{{\left(\partial E/\partial H_{\mathrm{int}}\right)}_{T}},$$

where $k_{B}$ is the Boltzmann constant, *T* is the temperature, and *E* is the thermal activation energy for magnetic viscosity. At low temperatures, the term $k_{B}\;T$ dominates and the parameter $S_{v}\left(H_{\mathrm{cJ}}\right)$ increases with increasing temperature up to a maximum of around 80 K for sintered FeNdB [9,15,30]. By contrast, the term ${\left(\partial E/\partial H_{\mathrm{int}}\right)}_{T}$ also increases monotonically with increasing temperature and becomes dominant for higher temperatures. As a result, the parameter $S_{v}\left(H_{\mathrm{cJ}}\right)$ for sintered FeNdB declines at temperatures higher than 80 K. From the spin reorientation temperature of Nd${}_{2}$Fe${}_{14}$B at around 135 K [31,32], $S_{v}$ decreases linearly [9,15,30] up to the temperature range between 303 and 433 K [10,16], as is also the case with the results for the $S_{v}\left(H_{D5}\right)$ presented in this study. It has also been shown in previous studies that ${\left(\partial E/\partial H_{\mathrm{int}}\right)}_{T}=J_{s}\;v$ [8,29], where $J_{s}$ is the saturation polarization of the material and *v* is the so-called activation volume where the thermally activated demagnetization process occurs in a hard magnetic grain by forming a domain wall. Substitution in Equation (6) then gives

(7) $$v=\frac{k_{B}\;T}{J_{s}\;S_{v}}.$$

**Figure 5:**
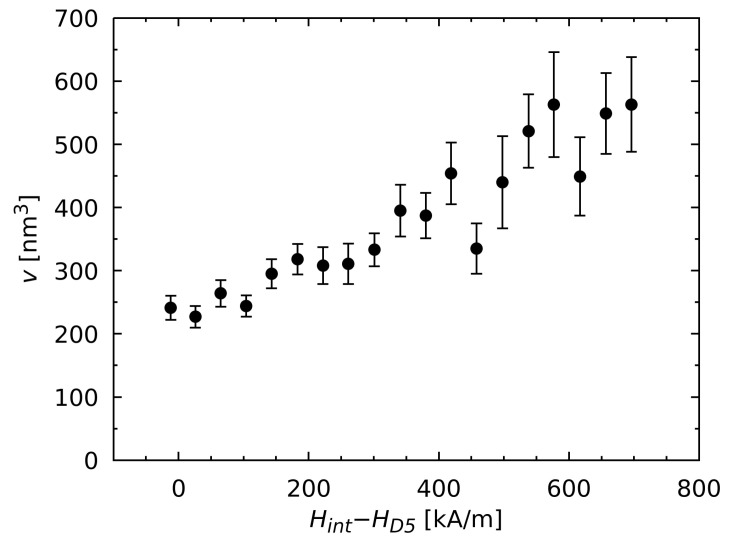
Activation volume *v* for the HREE-rich material M4 at 303 K, which is dependent on the internal magnetic field $H_{\mathrm{int}}$ with regard to the demagnetizing field $H_{D5}$. The values were calculated from the $S_{v}$ values in Figure 3d in accordance with Equation (7), where $J_{s}=1.24\;T$.

As an example, the activation volume for the material M4 at 303 K according to Equation (7) is shown in Figure 5. Due to the relation $v\propto 1/S_{v}$, *v* decreases with stronger applied fields. This behavior can be explained by the imperfect microstructure of the sintered material. The grains of the material exhibit regions at the grain boundary where the hard magnetic properties are lowered and the demagnetization is facilitated in the presence of an applied opposing field [32,33]. Due to this fact, grains with larger regions of defects, i.e., where the demagnetization of the grain begins within the activation volume, are already demagnetized at weaker applied fields than in grains with smaller regions of magnetic defects. As a consequence, the coercivity $H_{\mathrm{cJ}}$ of the entire material as an average over all consisting grains is much lower than theoretically expected. This phenomenon is known as Brown’s paradox, which has been used as an explanation at the theoretical level by different studies, e.g., by Kronmüller [34] or by Givord et al. [30]. The correlation between the parameter $S_{v}$ and the coercivity can therefore be observed not only between the different magnetic materials (like those shown in Figure 3), but also within a material consisting of grains with different-sized defected regions and thus activation volumes. With stronger internal fields, grains with smaller regions of defects and thus larger coercivities and $S_{v}$ values preferentially demagnetize. $S_{v}$ therefore increases tendentially as the opposing field becomes stronger.

## 6. Conclusions

In this study, commercial, sintered FeNdB magnets of different magnet grades were investigated with regard to their time-dependent aging, which is also called magnetic viscosity, in the industrially relevant magnetic fields and temperature ranges. The investigations led to the following conclusions:

- The magnetic viscosity parameter $S_{v}$ as a measure for the time-dependent aging of a magnet at an opposing magnetic field increases with stronger internal fields up to the industrially relevant demagnetizing field $H_{D5}$. The reason for the field dependence of $S_{v}$ in sintered FeNdB magnets can be explained by the influence of the microstructure, which consists of hard magnetic grains with different magnetic stabilities.
- The parameter $S_{v}$ increases with increasing coercivity $H_{\mathrm{cJ}}$ for the FeNdB materials of different magnetic grades.
- The parameter $S_{v}$ of a sintered FeNdB magnet in the region of the demagnetizing field $H_{D5}$ approximately decreases linearly with increasing temperature. However, the decrease in case of the HREE-rich material is very weak.
- A difference with regard to the parameter $S_{v}$ for both the materials with similar coercivity and temperature dependence but different types of processing could not be found. However, the question as to whether the different nature of the grain boundaries caused by GBDP have no influence at all on the magnetic viscosity must be shown by further investigations on more FeNdB magnets with similar magnetic properties but different processing procedures/parameters.

The parameter $S_{v}$ of FeNdB magnets strongly correlates with the coercivity $H_{\mathrm{cJ}}$, which in turn is influenced by various parameters. Therefore, the external factors that cause a decrease in the average coercivity of the magnetically metastable grains of a magnet (e.g., microstructures with lower amounts of HREEs or a coarser grain structure, higher temperatures, or stronger opposing magnetic fields) also lead to lower values of $S_{v}$. The results of this study allow designers of electrical components to estimate the temporal magnetic losses of FeNdB magnets under real operating conditions in a better way. For instance, different aging scenarios are shown in Table 4 for the material M2, which was aged at 373 K for 10 years. When the material M2 was exposed to an internal field $H_{\mathrm{int}}$ at −640 kA/m, which is about 100 kA/m weaker than $H_{D5}$, the magnetic viscosity caused a decrease in the polarization of about 12%. On the other hand, the aging of the same material at a weaker field of −440 kA/m only led to a negligible loss of polarization of about 1%. As a result, for the machine design and manufacturing process, more cost-effective magnetic materials that are still sufficient for guaranteeing the performance requested for the whole lifetime of the component can be chosen.

## Figures and Tables

**Table 1:** Overview of the investigated commercially available FeNdB magnets with the amounts of their most important ingredients, the mean grain size, and their magnetic properties. The proportion of other metallic additives (Co, Cu, and Ga) was between 1.1 and 1.4 at.% for all listed materials.

Material	Amount of Element (at.%)					Mean Grain	Sintering	$H_{\mathrm{cJ}}$	$B_{r}$
	Nd	Pr	Tb	Dy	Fe	Size (µm) ^a^	Processing	(kA/m) ^b^	(T) ^b^
M1	11.0	3.5			78.6	5.7	conventionally	1085	1.46
M2	10.9	2.6	0.6		79.2	5.8	conventionally	1615	1.41
M3	10.9	3.5	0.3		78.8	5.9	additional GBDP ^c^	1737	1.47
M4	11.4	3.5		5.2	76.7	11.6	conventionally	2954	1.16

^a^ Determined by means of electron backscattering diffraction (EBSD). ^b^ Measured by means of vibrating sample magnetometer (VSM) at 293 K. ^c^ Grain Boundary Diffusion Process.

**Table 2:** Demagnetizing field $H_{D5}$ of the investigated FeNdB materials determined according to [23] at selected temperatures.

T (K)	$H_{D5}$ (kA/m)			
	M1	M2	M3	M4
303	−879	−1395	−1513	−2632
343	−565	−994	−1092	−2060
373	−399	−739	−823	−1684
403	−283	−532	−602	−1328
433		−367	−423	−1012

**Table 3:** Slope of the change of the magnetic viscosity parameter $S_{v}$ at the demagnetizing field $H_{D5}$ in dependence of the temperature *T* calculated from the linear regression of the values in Figure 4.

Material	$dS_{v}\left(H_{D5}\right)/dT$ (%/K)
M1	−0.45±0.03
M2	−0.29±0.04
M3	−0.30±0.06
M4	−0.05±0.03

**Table 4:** Exemplary aging scenarios for the material M2 when exposed to a constant internal magnetic field at 373 K for 10 years ($\ln(t/t_{0})\approx 20$). The magnetic viscosity of the material leads to an “effective” internal field $H_{\mathrm{int}}^{\mathrm{eff}}=H_{\mathrm{int}}+\Delta H$ that is in accordance with Equation (2) and thus to a decrease $\Delta J=J\left(H_{\mathrm{int}}^{\mathrm{eff}}\right)-J\left(H_{\mathrm{int}}\right)$ of the magnetic polarization after 10 years.

$H_{\mathrm{int}}$ (kA/m)	$S_{v}$ (kA/m)	ΔH (kA/m)	$H_{\mathrm{int}}^{\mathrm{eff}}$ (kA/m)	ΔJ (%)
−640	7	−140	−780	−12.0
−440	4	−80	−520	−1.1

## Data Availability

Data are contained within the article.

## References

[1] Cui J., Ormerod J., Parker D., Ott R., Palasyuk A., Mccall S., Paranthaman M.P., Kesler M.S., McGuire M.A., Nlebedim I.C. (2022). Manufacturing Processes for Permanent Magnets: Part I–Sintering and Casting. JOM.

[2] Collocott S.J. (2016). Magnetic Materials: Domestic Applications. Reference Module in Materials Science and Materials Engineering.

[3] Villani M. High Performance Electrical Motors for Automotive Applications–Status and Future of Motors with Low Cost Permanent Magnets. Proceedings of the 8th International Conference on Magnetism and Metallurgy.

[4] Sugimoto S., Fujisaki K. (2019). History and Future of Soft and Hard Magnetic Materials. Magnetic Material for Motor Drive Systems.

[5] Müller M., Harada H., Warlimont H., Martienssen W., Warlimont H. (2018). Magnetic Materials. Springer Handbook of Materials Data.

[6] Barbier J.C. (1954). Le traînage magnétique de fluctuation. Ann. Phys..

[7] Street R., Woolley J.C. (1949). A Study of Magnetic Viscosity. Proc. Phys. Soc. Lond. Sect. A.

[8] Street R., Day R.K., Dunlop J.B. (1987). Magnetic viscosity in NdFeB and SmCo_5_ alloys. J. Magn. Magn. Mater..

[9] Givord D., Tenaud P., Viadieu T., Hadjipanayis G. (1987). Magnetic viscosity in different Nd-Fe-B magnets. J. Appl. Phys..

[10] Nishio H. (1988). Magnetic aftereffect of Nd-Fe-B sintered magnets. IEEE Trans. Magn..

[11] Villas-Boas V., Missell F.P., Schneider G., Lu Q., Givord D. (1990). Coercivity and magnetic viscosity in Nd_80_Fe_15_B_5_. Solid State Commun..

[12] Becher M., Seeger M., Bauer J., Goll D., Kronmüller H., Schultz L., Müller K.H. (1998). Magnetic viscosity measurements on nanocrystalline NdFeB and PrFeB magnets. Proceedings of the 10th International Symposium on Magnetic Anisotropy and Coercivity in Rare-Earth Transition Metal Alloys.

[13] Grössinger R., Turtelli Sato R., Téllez-Blanco C. (2004). The influence of the magnetic viscosity on pulsed field measurements. J. Optoelectron. Adv. Mater..

[14] Street R., Woolley J.C., Smith P.B. (1952). Magnetic Viscosity under Discontinuously and Continuously Variable Field Conditions. Proc. Phys. Soc. Lond. Sect. B.

[15] Givord D., Lienard A., Tenaud P., Viadieu T. (1987). Magnetic viscosity in Nd-Fe-B sintered magnets. J. Magn. Magn. Mater..

[16] Nishio H. (1990). Magnetic Aftereffect Constant of Nd-Fe-B Sintered Magnets. IEEE Transl. J. Magn. Jpn..

[17] Haavisto M., Paju M. (2009). Temperature Stability and Flux Losses Over Time in Sintered Nd–Fe–B Permanent Magnets. IEEE Trans. Magn..

[18] Haavisto M., Tuominen S., Santa-Nokki T., Kankaanpää H., Paju M., Ruuskanen P. (2014). Magnetic Behavior of Sintered NdFeB Magnets on a Long-Term Timescale. Adv. Mater. Sci. Eng..

[19] Street R., Woolley J.C. (1950). Time Decrease of Magnetic Permeability in Alnico. Proc. Phys. Soc. Lond. Sect. B.

[20] Néel L. (1950). Théorie du traînage magnétique des substances massives dans le domaine de Rayleigh. J. Phys. Radium.

[21] Néel L. (1951). Le traînage magnétique. J. Phys. Radium.

[22] Aharoni A. (1998). Demagnetizing factors for rectangular ferromagnetic prisms. J. Appl. Phys..

[23] (2015). Magnetic ,Materials – Part 8-1: Specifications for Individual Materials – Magnetically Hard Materials.

[24] Givord D., Tenaud P., Viadieu T. (1986). Analysis of hysteresis loops in Nd-Fe-B sintered magnets. J. Appl. Phys..

[25] Nishio H., Yamamoto H., Nagakura M., Uehara M. (1990). Effects of machining on magnetic properties of Nd-Fe-B system sintered magnets. IEEE Trans. Magn..

[26] Katter M., Üstüner K., Blank R. (2006). Model for Calculating *J*(*H*) Curves of Ni Coated Nd-Fe-B Magnets. J. Iron Steel Res. Int..

[27] Volegova E.A., Andreev S.V., Selezneva N.V., Urzhumtsev A.N., Volegov A.S. (2019). Effect of intergrain exchange interaction on magnetic viscosity of nanocrystalline isotropic NdFeB magnets. J. Phys. Conf. Ser..

[28] Wohlfarth E.P. (1984). The coefficient of magnetic viscosity. J. Phys. F Met. Phys..

[29] Gaunt P. (1986). Magnetic viscosity and thermal activation energy. J. Appl. Phys..

[30] Givord D., Tenaud P., Viadieu T. (1988). Coercivity mechanisms in ferrites and rare earth transition metal sintered magnets (SmCo_5_, Nd-Fe-B). IEEE Trans. Magn..

[31] Givord D., Li H.S., de la Bâthie R.P. (1984). Magnetic properties of Y_2_Fe_14_B and Nd_2_Fe_14_B single crystals. Solid State Commun..

[32] Kronmüller H., Fähnle M. (2003). Micromagnetism and the Microstructure of Ferromagnetic Solids.

[33] Goll D., Kronmüller H., Parkin S. (2007). Micromagnetism–Microstructure Relations and the Hysteresis Loop. Handbook of Magnetism and Advanced Magnetic Materials.

[34] Kronmüller H. (1987). Theory of Nucleation Fields in Inhomogeneous Ferromagnets. Phys. Status Solidi B.

